# War

**Published:** 1876-06

**Authors:** 


### Its Hardships.

IN its various developments, war is ever fruitful of hardships unavoidable, terrible; and of cruelties which in countless cases, must necessarily fall alike upon the just and the unjust. During the Indian wars of the New World, commencing with the famous chief, King Philip, and continuing for nearly a century, the appalling instances of heartless cruelties are abundant. The grievances of that terrible struggle (tending to the extermination of the one race or the other) fell upon the guiltless heads and hearts of the New England English mothers of that day *more heavily* than upon any other class;—*women* whose faith, endurance, and valor have never been equaled in the world's history. True it is:

From war-whoops wild and earth in crimson glow,
A wail went up, a note of woman's woe,
Fierce vengeance tempts her singleness of heart,
Her heroism true, her guileless art;
Her purity, her own maternal care,
Her faith in God, that never knows despair;
Her love, indeed, that triumphs most and best
In trial sad, when most by danger pressed;

Whose truth endures when fails our vital breath,
Inspires fond hope, and smooths the bed of death.
Such were the hearts whose wails went up afar,
That brooked the fury of King William's war;
Whose just protection savages defied,
And dearest hopes of house and home denied;
Around her hearth from hidden ambush springs
The lurking foe, and death with honor brings.

The heroism of many of the primeval mothers of New England will never be forgotten. Not long since a monument was erected to their memories, the heroic Hannah Duston of the Contoocook being taken as a true representative of the peerless valor which inspired them at that period.

And this is war; and such in wrath makes haste
To lay the white man's cot and village waste;
That deals in daggers poisoned, coated o'er,
The fagot torch, and gluts on human gore.
Against such crime the settlers strong unite;
In divers ways they rally for the fight;
Some seek defense by force of gun and dogs,
Some take to garrisons strong built of logs;
And some in squads with weapons rude assail
The foe, and fierce pursue the hidden trail.

Among the like hardships we have a further illustration in the conflict at arms between General Sherman and the Confederate General Hood at Atlanta, Ga.

Up to the 20th of July, 1864, Hood with a large army had been intrenched there, and Sherman (with his gallant field marshals, Hooker, McPherson, Scofield, Johnson, Palmer, Blair, McCook, Stoneman, Logan, Kilpatrick, Wheeler, Thomas, Couch, Davis, Howard, and perhaps other generals), commanding an army still larger, had from afar victoriously been advancing upon Atlanta, and arriving, had come to a stand within five miles of the breastworks of Hood's lines. Then came the "tug of war." Hood not waiting to be besieged, sallying forth offensively, gave battle, and on five or six days the thunders of contending armies muttered; and then and there thousands perished. Hood, on the whole getting the worst of it, retreated between two days, and intrenched his army some thirty miles away. Sherman, of course, advancing held the city, and by a letter to Hood proposed the removal of its inhabitants to some other locality out of harm's way; and to that end tendered the employment of his own army wagons, and gave to Hood and the city their choice as to the locality to which they should be removed, to be cared for by him.

To this proposition Hood, of course, acceded, *but demurred to it;* and, as if to create a national indignation against Sherman, denounced the *order* as "*unprecedented*, *studied*, and *ungenerous cruelty*."

To this Sherman in a letter replied; and, in substance, maintained his Order to be, under all the circumstances, humane; and Hood's complaint hypocritical. This rejoinder brought back a petition from the city of Atlanta, through its city council, wherein Sherman was *entreated*, in the name of humanity, to desist from the enforcement of the Order, which to them appeared most oppressive and cruel.

To this petition Sherman, giving reasons for his order, and moralizing as if to suggest, that *they who begin war* should be the last to complain of its hardships, answers nobly, and justly worthy to to be copied, as follows: *

"ATLANTA, GA., *September* 12, 1864.

"JAMES M. CALHOUN, *Mayor*, E. E. RAWSON, *and* S. C. WELLS, *representing the City Council of Atlanta:*

"GENTLEMEN—I have your letter of the 11th, in the nature of a petition, to revoke my orders removing all the inhabitants from Atlanta. I have read it carefully, and give full credit to your statements of the distress that will be occasioned by it, and yet shall not revoke my order, simply because my orders are not designed to meet the humanities of the case, but to prepare for the future struggles, in which millions, yea hundreds of millions, of good people outside of Atlanta have a deep interest. We must have *Peace*, not only at Atlanta, but in all America.

"To secure this, we must stop the war that now desolates our once happy and favored country. To stop war, we must defeat the rebel armies that are arrayed against the laws and Constitution, which all must respect and obey. To defeat these armies we must prepare the way to reach them in their recesses, provided with the arms and instruments which enable us to accomplish our purpose.

"Now, I know the vindictive nature of our enemy, and that we may have many years of military operations from this quarter, and, therefore, deem it wise and prudent to prepare in time. The use of Atlanta for warlike purposes is inconsistent with its character as a home for families.

"There will be no manufactures, commerce, or agriculture here for the maintenance of families, and, sooner or later, want will compel the inhabitants to go. Why not go *now*, when all the arrangements are completed for the transfer, instead of waiting till the plunging shot of contending armies will renew the scene of the

* See Headley's History, Vol. II., p. 342.

past month? Of course, I do not apprehend any such thing at this moment, but you do not suppose that this army will be here till the war is over. I can not discuss this subject with you fairly, because I can not impart to you what I propose to do; but I assert that my military plans make it necessary for the inhabitants to go away, and I can only renew my offer of services to make their exodus in any direction as easy and comfortable as possible. You can not qualify war in harsher terms than I will.

"War is cruelty, and you can not define it; and those who brought war on our country deserve all the curses and maledictions a people can pour out. I know I had no hand in making this war, and I know I will make more sacrifices to-day than any of you to secure peace. But you can not have peace and a division of our country. If the United States submits to a division now, it will not stop, but will go on till we reap the fate of Mexico, which is eternal war.

"The United States does and must assert its authority wherever it has power; if it relaxes one bit to pressure, it is gone, and I know that such is not the national feeling. This feeling assumes various shapes, but always comes back to that of *Union*. Once admit the Union, once more acknowledge the authority of the National Government, and instead of devoting your houses and streets and roads to the dread uses of war, I and this army become at once your protectors and supporters, shielding you from danger, let it come from what quarter it may. I know that a few individuals can not resist a torrent of error and passion, such as has swept the South into rebellion; but you can point out, so that we may know those who desire a government, and those who insist on war and its desolation.

"You might as well appeal against the thunder-storm as against these terrible hardships of war. They are inevitable; and the only way that the people of Atlanta can hope once more to live in peace and quiet at home, is to stop this war, which can alone be done by admitting that it began in error and is perpetuated in pride. We don't want your negroes or your horses, or your houses or your land, or anything you have; but we do want, and will have a just obedience to the laws of the United States.

"That we will have, and if it involves the destruction of your improvements, we can not help it. You have heretofore read public sentiment in your newspapers, that live by falsehood and excitement, and the quicker you seek for truth in other quarters, the better for you.

"I repeat, then, that, by the original compact of government, the United States had certain rights in Georgia, which have never been relinquished, and never will be; that the South began war by seizing forts, arsenals, mints, custom-houses, etc., etc., long before Mr. Lincoln was installed, and before the South had one jot or tittle of provocation. I, myself, have seen in Missouri, Kentucky, Tennessee, and Mississippi, hundreds and thousands of women and children fleeing from your armies and desperadoes, hungry and with bleeding feet. In Memphis, Vicksburg, and Mississippi we fed thousands upon thousands of the families of rebel soldiers left on our hands, and whom we could not see starve. Now that war comes home to you, you feel very different; you deprecate its horrors, but did not feel them when you sent carloads of soldiers and ammunition, and moulded shell and shot, to carry war into Kentucky and Tennessee, and desolate the homes of hundreds and thousands of good people, who only asked to live in peace at their old homes, and under the Government of their inheritance.

"But these comparisons are idle. I want peace, and believe it can only be had through Union and war, and I will ever conduct war purely with a view to perfect and early success.

"But, my dear sirs, when that peace does come, you may call on me for anything. Then will I share with you the last cracker, and watch with you to shield your homes and families against danger from every quarter.

"Now, you must go, and take with you the old and feeble; feed and nurse them, and build for them, in more quiet places, proper habitations to shield them against the weather, until the mad passions of men cool down, and allow the Union and peace once more to settle on your old homes at Atlanta.

"Yours, in haste, W. T. SHERMAN,

"*Major-General.*"

